# Scaling up integrated care for HIV and other chronic conditions in routine health care settings in sub-Saharan Africa: Field notes from Uganda

**DOI:** 10.5334/ijic.6962

**Published:** 2023-08-11

**Authors:** Faith Moyo, Josephine Birungi, Anupam Garrib, Ivan Namakoola, Joseph Okebe, Sokoine Kivuyo, Gerald Mutungi, Sayoki Mfinanga, Moffat Nyirenda, Shabbar Jaffar

**Affiliations:** 1 Department of International Public Health, Liverpool School of Tropical Medicine, Liverpool, United Kingdom; 2 Medical Research Council/Uganda Virus Research Institute and London School of Hygiene and Tropical Medicine Uganda Research Unit, Entebbe, Uganda; 3 The AIDS Support Organization, Kampala, Uganda; 4 Department of Clinical Sciences, Liverpool School of Tropical Medicine, Liverpool, United Kingdom; 5 Muhimbili Medical Research Centre, National Institute for Medical Research Muhimbili Research Centre, Dar Es Salaam, Tanzania; 6 Non-Communicable Diseases Control Programme, Ministry of Health, Kampala, Uganda; 7 Faculty of Epidemiology and Public Health, London School of Hygiene and Tropical Medicine, London, United Kingdom

**Keywords:** integrated care, non-communicable diseases, sub-Saharan Africa, HIV, chronic care

## Abstract

**Introduction:**

Integration of HIV and non-communicable disease (NCD) services is proposed to increase efficiency and coverage of NCD care in sub-Saharan Africa.

**Description:**

Between October 2018 to January 2020 in Tanzania and Uganda, working in partnership with health services, we introduced an integrated chronic care model for people with HIV, diabetes and hypertension. In this model, patients were able to access care from a single point of care, as opposed to the standard of siloed care from vertical clinics. When the study ended, routine clinical services adopted the integrated model. In this article, we discuss how the model transitioned post hand-over in Uganda and draw lessons to inform future scale-up.

**Discussion:**

The findings suggest potential for successful uptake of integrated chronic care by routine clinical services in sub-Saharan Africa. This approach may appeal to health care service providers and policy makers when they can quantify benefits that accrue from it, such as optimal utilization of health resources. For patients, integrated care may not appeal to all patients due to HIV-related stigma. Key considerations include good communication with patients, strong leadership, maintaining patient confidentiality and incorporating patient needs to facilitate successful uptake.

**Conclusion:**

Evidence on the benefits of integrated care remains limited. More robust evidence will be essential to guide scale-up beyond research sites.

## (1) Introduction

The height of the HIV epidemic in the mid-1980s weighed heavily on health systems in sub-Saharan Africa to a point of near collapse. The epidemic rose quickly, with HIV-related deaths peaking at over 2 million deaths a year on the continent in the early 2000s [1]. Two decades on, and the tide has turned. Challenges remain but HIV has become a manageable chronic condition owing to intensive disease control measures, with life expectancy now close to that of people who do not have HIV in sub-Saharan Africa. For example, life expectancy after starting ART at ages 20 and 35 years old is an additional 28 and 25 years respectively, with projections higher for females than males in each case [2,3]. As more people are ageing with HIV, sub-Saharan Africa is seeing a rapid rise in non-communicable diseases (NCDs) both in people living with HIV and the general population. About 2 million premature deaths annually are now attributed to diabetes and hypertension alone [4]. It is estimated that the burden of NCDs in the region will overtake the combined burden of all infectious diseases in the next decade [4,5]. Health care systems from sub-Saharan Africa are strained for resources and re-organisation may help them to cope with the burden of chronic conditions [6,7].

HIV control programmes in sub-Saharan Africa operate in silos. When the provision of antiretroviral therapy was first scaled-up, it was funded largely by international donors and through a separate funding mechanism [8]. Today, about 60% of HIV programmes are funded from domestic sources [9] but the HIV control programmes remain stand-alone and separate from other parts of the health system. Non-communicable disease control and care programmes on the other hand are relatively new in Africa and are characterized by significant gaps in funding and implementation [7]. Currently, NCDs are managed in other parts of the health system, with less than 10% of people with either hypertension or diabetes estimated to be in regular care [7,10,11,12].

HIV and NCDs have similar attributes. They are chronic conditions, which require long term adherence to medicines, ongoing clinical care and monitoring. In principle, NCD programmes could leverage experience from and share resources with HIV programmes to strengthen NCD care [13,14,15] but the evidence of the benefits and potential harms of integrated chronic care in routine African settings is sparse.

Between 5 October 2018 and 30 January 2020, we conducted a prospective study evaluating the feasibility of integrated chronic care for HIV, diabetes and hypertension in 10 health care facilities offering primary health care services in urban and peri-urban areas in Tanzania and Uganda [15]. The study demonstrated high levels of retention (>80%) in integrated care for people living with either HIV or NCDs, or combinations of these conditions [15]. Research involving health systems interventions requires major changes to health services delivery for research purposes. In our case, each health facility manager re-organised clinics in their health facility from delivering stand-alone care to a single integrated care clinic. The study was a small feasibility study, designed to inform a much larger trial, and analyses and interpretation of data took many months. Thus, at the end of the follow-up of participants, definitive research evidence was not available to health care managers. Knowledge on what happens in clinical practice after such studies end is limited and, in this article, we discuss how clinical practice in relation to the integrated chronic care approach evolved after the study ended and draw lessons to inform future scale-up.

## (2) Overview of study methodology

A detailed description of the methodology for the study has been published elsewhere [15]. Prior to the study, patients with diabetes, hypertension or HIV were managed in stand-alone clinics irrespective of gender or whether they had one or more of these conditions.

Working in partnership with routine clinical services, we set up an integrated chronic care clinic to provide care to patients with either HIV, diabetes or hypertension or a combination of these conditions, from a single point of service. The integrated chronic care clinic comprised about 250–300 patients at each facility. It was run alongside the vertical care clinics which continued to provide stand-alone care routinely. The study was conducted in five clinics each in Tanzania and Uganda over a period of 16 months, between 5 October 2018 and 30 January 2020. When the research study ended, follow-up of the participants was ended, and they were free to return to the standard care model of vertical service provision.

The findings from the study were shared with policy makers and health facility staff some months after the study ended. As this was a feasibility study, a larger trial (now underway) was needed to provide more robust evidence for policy considerations [16]. However, immediately after the study ended, facility level health care managers in Uganda wanted to implement integrated care for all patients as they had witnessed its benefits and were convinced that the evidence from the pilot study was sufficient to start the integration, pending further evidence from the larger study. This was not possible in Tanzania, where the Ministry of Health required additional evidence before approving modifications to health care delivery.

In Uganda, a decentralised management model and full support from the Ministry of Health, enabled government managers of the four public health facilities (Kiswa, Kisugu, Ndejje and Wakiso) that were part of the study, to adopt and scale-up the integrated care model for all patients attending for HIV, diabetes and hypertension care. They took the initiative independently without influence from the research team and without consultation amongst themselves. The research team played an advisory role, only when required and were not directly involved in the operations of clinics.

The four sites were government primary health care facilities, providing services in urban and peri-urban areas located in Kampala, Uganda. The sites included a mix of regional (Ndejje and Wakiso) and small health centres (Kiswa and Kisugu). Prior to integration, each facility provided HIV services from stand-alone HIV clinics. HIV clinics had their own reception, waiting areas, dedicated clinical staff and separate pharmacies. In the larger facilities, diabetes and hypertension services operated from separate clinics, while in the smaller facilities patients seeking diabetes and hypertension services were seen in outpatient clinics [15]. The staff compliment providing integrated care included nurses and medical officers, employed under local clinical practice according to national guidelines [15]. Table 1 further summarizes the features of the four government sites.

**Table 1:** Evolution of the integrated chronic care model during scale-up in routine clinical services in Uganda (n = 4 facilities).


FEATURE	PRACTICE BEFORE PILOT STUDY *(STANDARD CARE)*	PILOT INTERVENTION *(STUDY CONDITIONS)*	PRACTICE AFTER THE PILOT STUDY *(UP TO ONE YEAR AFTER STUDY ENDED)*

*Reception, triage and waiting areas*	Reception, triage and waiting areas were separate for patients with HIV and NCDs across the sites. In 2/4 facilities, patients with NCDs did not have a designated NCD clinic but were seen alongside acute care in outpatient clinics.	Reception and triage areas for HIV and NCD clinics were combined, so that all patients were seen at a single reception and triage point. Patients with any condition also waited together.	Kiswa, Wakiso and Ndeje facilities maintained joint reception, triaging, and waiting areas at a single point for all patients regardless of medical condition. Kisugu shared reception and triage areas but separated waiting areas for patients with HIV and NCDs

*Medical records*	All sites had robust systems for capture and storage of HIV records, whereas systems for capture and storage of medical records for NCDs were informal or did not exist. For example, prescriptions for NCDs were documented in hand-held government medical forms or in patients’ books, which often got lost or they forgot to bring them when seeking care	HIV clinics used an HIV clinic card for documenting clinical information. An NCD clinic card similar to that for HIV was created for patients with NCDs. The same filing system used for HIV was used for NCDs and records for both conditions were stored together	Record capture and storage was maintained for HIV as part of standard care. For NCDs, all facilities continued with NCD record capture and filing system. However, NCD records were stored in separate rooms from HIV records. In Kisugu, the NCD care card was inserted into the HIV file for patients with HIV and other comorbidities

*Consultation*	Patients with HIV or NCDs were seen by different clinicians, either on different days or at different health facilities. Patients with HIV with concurrent conditions were referred to other health facilities to seek care for their conditions, resulting in multiple visits to the clinic for each condition In two facilities, without designated NCD clinics, patients with NCDs were managed alongside acute care	All patients regardless of condition were seen by the same clinicians. If a patient had more than one condition, management of both conditions occurred within the single consultation	Patients with HIV or NCDs continued to be managed by the same clinical staff. All conditions were managed during a single consultation across the facilities

*Patient tracking and follow up*	HIV clinics had robust structures for patient follow up and tracking, with clear appointment record books and/or electronic registers to track patient visits. Patients with NCDs were not followed up, patients presented for care when they wished, alongside episodic care	Follow up of patients with NCDs was aligned with systems for tracking and follow up of patients with HIV	Kiswa, Kisugu and Wakiso facilities continued tracking and following up patients with HIV or NCDs the same way. When patients missed scheduled visits, phone calls were made to remind them of clinic visits. Home visits were made but for patients with HIV only. At Ndejje, patients with NCDs were not followed up. The prerogative to honour scheduled clinic visits was left to the patient. Only patients with HIV and other comorbidities were followed up

*Pharmacy and dispensing*	Separate pharmacies were used for patients with HIV or NCDs. Where patients shared a pharmacy, separate dispensing points were used for HIV and NCDs. For example, NCD drugs were dispensed at the front window and the HIV drugs were dispensed at the back window. Drugs were also dispensed by different pharmacists	The same pharmacy with same dispensing point (or adjacent dispensing points) for HIV and NCD drugs was used. Staff received training to be able to dispense drugs for either condition so that patients only had to go to a single window to receive their drugs	Both patients with HIV and NCDs continued getting their drugs from the same pharmacy, sharing dispensing staff. Kiswa, Ndejje and Wakiso facilities dispensed both HIV and NCD drugs from the same area while in Kisugu separate dispensing areas were used for either condition

*Counselling and patient education*	Patients with HIV received adherence counselling while counselling services were not available for patients with NCDs	Counselling services were aligned so that all patients were receiving the same care package	Counselling services were maintained for both groups of patients at Ndejje and Kiswa health facilities. In Wakiso and Kisugu, patients with NCDs and those with HIV were counselled by different clinical staff

*Laboratory*	Patients with HIV and NCDs received the same centralized laboratory services	Patients with HIV and NCDs received the same laboratory services, however during the study fasting diabetic patients were prioritised. The study also provided free glucose strips for monitoring blood glucose among patients with diabetes	Blood sugar measurements for patients with diabetes, regardless of HIV status were taken at the NCD triage across the facilities. All other samples were sent to centralized laboratory services for clinical investigations


While this was possible among the four Ugandan public health facilities, the fifth site (The Aids Support Organization, TASO) was a non-governmental organization which specialised in HIV care and was unable to take up integration as this required extensive operational changes to its care delivery and funding models.

The remainder of the article describes how the integrated chronic care approach evolved in the four Ugandan government facilities and some of the challenges that were faced. We relied on direct observation to describe how the integrated care approach evolved in routine clinical services after the research study. We observed and documented how operations of the integrated care clinics were modified pre- and post the research study. The research team was invited to multiple stakeholder meetings (patients, facility managers and policy makers) to discuss progress and challenges faced by the integrated care clinics. We documented minutes at these gatherings across the health facilities. We captured information from patient representatives on patients’ experiences when accessing care in integrated care clinics. We also compiled reports from health facility managers detailing challenges and benefits of scaling up integrated care from the same stakeholder meetings or through key informant interviews where possible.

## (3) Evolution of the integrated chronic care approach post-handover to clinical services

### (3.1) Patient engagement and empowerment

Integrated chronic care services involve patients with different health conditions using the same triage and waiting areas, being managed by the same clinical team and receiving medications from the same pharmacy [17]. When this was implemented in a research setting, patients received information about the impending change and had opportunity to ask questions and discuss issues. When clinical services scaled up integrated chronic care into routine services to all patients, such empowerment of patients will not have been done at the outset and health facility managers noted that some patients living with HIV expressed concerns around disclosure. Health facility managers reported that a few patients attended the clinic disguised heavily with face coverings or requested to have their drugs delivered to them. Some patients with NCDs were also uncomfortable mixing with patients with HIV and feared being perceived as HIV positive.

To tackle this, health facility managers held regular meetings with patient representatives and policy makers explaining the need for and benefits of the integrated care model. Patients were made to feel that their concerns were heard, and that their input would be incorporated where possible to avoid discomfort. The research staff were also included in these meetings to provide support to health services on technical aspects of the roll-out of integrated care. Table 1 describes some of the temporary modifications to the integrated chronic care model adopted by routine clinical services to accommodate their requirements in terms of patient needs, staff, space and other considerations, while awaiting policy provision for further scale-up of the model.

### (3.2) Medicines availability and drug supply for NCDs

Probably the biggest challenge faced by routine health services was the limited availability of medicines for diabetes and hypertension. Medicines supply for first-line drugs for hypertension and diabetes was strengthened during the feasibility study, but this could not be sustained once the study ended. Health facility managers reported that the supply of NCD drugs was erratic across the clinics. During drug shortages, patients with NCDs had to pay for drugs out-of-pocket or via health insurance if they had this.

Following a circular from the Ugandan Ministry of health that provided clearance for patients to form NCD support clubs for counselling and adherence support, health facility managers invited patients to a large public meeting to facilitate formation of NCD clubs among patients living with NCDs to improve adherence to treatment and retention in care.

In the meeting, an NCD club committee was elected to coordinate operations of the club. As part of strengthening adherence, patients agreed to contribute small amounts of money to a pool for purchase of NCD drugs when facilities ran out of supply. The committee was tasked with scheduling future meetings, collecting financial contributions, managing records of members, and facilitating drug purchases on behalf of the club. Each member contributes approximately £3–£5 monthly into a central bank account and the pooled funds are then used by the NCD club committee for bulk purchase of drugs at substantially subsidised costs, usually less than 50% of the costs of drugs available through private pharmacies. Drugs belonging to the club are stored in health centre pharmacies but separate from the government stocks of drugs.

During government NCD drug shortages, the drugs belonging to the NCD club were dispensed through the hospital or health centre pharmacies by government pharmacists working with the NCD club committee to club members only. However, some patients could not afford the £3–£5 monthly contributions. In some clinics, facility managers secured external funding (from churches, politicians, and philanthropists) to purchase NCD drugs to support patients who had no money.

### (3.3) Healthcare staff capacities and patient flows

The routine integrated chronic care clinics also experienced staffing issues initially. Some health workers did not have sufficient skills to manage both HIV and NCDs. Health facility managers reported that consultations took longer, and patients experienced longer waiting periods for care. This was because the integrated chronic care model requires clinicians to manage multiple conditions in a single consultation, yet some health workers had limited skill sets.

In other facilities, clinics were congested in the beginning because of poor patient flows and long queues for care. In turn, patients were not happy with the delivery of services when integrated care was first rolled out. Health facility managers also reported that some staff became apprehensive about work overload when integrated care was first introduced. For example, the introduction of an NCD care card, the clinical care record for people with NCDs, was perceived as extra workload by some staff. Other clinical staff were reluctant to provide integrated services due to their limited skill set and would refer patients to a more qualified clinician. This did not sit well with patients who had been waiting for care in long queues. For staff, this meant that individuals with expanded clinical focus were overloaded with patients initially.

With time, more staff underwent refresher training on management of diabetes, hypertension and HIV. Specialist physicians were also available in the facilities to provide mentorship and support to junior staff for complex medical cases. Patient flows were re-organised across the facilities. These efforts coupled with experience, capacitated routine staff to manage all three conditions in a single consultation with comparative ease. A year later, health facility managers reported that both patients and staff have fully embraced the integrated chronic care model and it remains popular with both parties.

## (4) Discussion

Our findings demonstrate potential for successful uptake of integrated chronic care by routine clinical services in sub-Saharan Africa. Findings suggest that this model of care will be popular among health care providers and policy makers, particularly when they can quantify the benefits that could accrue from this model of care, such as optimal utilization of health resources and reduction of costs associated with health care delivery. At present, rigorous evidence on these benefits, which will be central to convincing health care providers, patients and other stakeholders, remains limited. Our findings also reveal stigma and discrimination as well as health system challenges as potential threats to successful scale up of integration if neglected. We discuss lessons learned for both health systems and patients and highlight gaps for further research to inform future scale up of integrated chronic care in limited resource settings.

### Lesson 1. Task re-distribution within the health care workforce is feasible in African settings and optimizes the use of available human resources

Staff shortages and exodus of skilled personnel is common in routine clinical services across Africa [18]. The impact of staff shortages on quality and delivery of health services is further compounded by the traditional vertical care systems, which have created a workforce with a limited skillset that is primarily focused on infectious diseases. This now needs changing due to the growing burden of NCDs in Africa.

To meet demand for multi disease care in the integrated clinics in this study, different cadres of clinical staff received continued training and mentorship to expand their skillset for target conditions. Health facility managers also re-distributed tasks among different staff in response to the expanded services required by patients in the integrated care clinics. For example, HIV clinicians not only focused on HIV care but were trained to screen for and manage hypertension and diabetes even in HIV negative clients. This maximized staff efficiency which can alleviate the impact of chronic staff shortages and human resource costs in African health care systems [19].

There was concern over the effect of task re-distribution on clinical and retention outcomes of the cohort receiving care in the integrated chronic care clinics. However, analysis of these outcomes in the feasibility study demonstrated good retention for all three study conditions and no negative effect on HIV control [15]. Other studies from Sub-Sahara Africa have demonstrated the feasibility of task-redistribution when integrating NCD care into existing HIV programmes in limited resource settings [17,20,21,22]. Future scale-up initiatives should consider investing in clear protocols and equitable allocation of roles and responsibilities to avoid overloading clinical staff. Further research should explore the utility and cost-effectiveness of an auxiliary/support staff model to supplement clinical personnel in high volume integrated chronic care clinics, as in the case of utilizing community health workers for HIV screening and linkage into care in HIV programmes.

### Lesson 2. Clear patient flows and organized health systems are central to efficiency of integrated care

Some of the facilities (Ndejje and Wakiso) that adopted the integrated model were high volume clinics. Congestion during peak hours was inevitable when integrated care was introduced. Longer wait times and a strained workforce had a temporary knock-on effect on quality of service and clients’ satisfaction. Health facility managers quickly re-organised patient flows in their clinics to suit their needs. Although the broader principles of integration were retained, health facility managers scaled up different models of care to accommodate their needs as demonstrated by Table 1. If governments decide to scale-up, health facility managers will need support and guidance from policymakers so that the scaled-up model of integrated care is broadly similar across different health facilities without compromising quality of care. Moreover, clear leadership within health facilities will be critical to enable steady transition from vertical to integrated care.

### Lesson 3. Microfinance schemes can address socioeconomic barriers to retention in integrated chronic care

In Uganda and other African countries, poor medicine supply for NCDs has probably been the major set-back to the effective control of NCDs [7]. People with limited ability to pay user-fees and purchase medicines for NCD care have a disproportionate risk of poor retention and suboptimal clinical outcomes. In this study, health care managers remarkably facilitated a system for patients to purchase medicines at substantially reduced costs. This demonstrates the importance of engaging with and empowering health managers and patients within research studies and provides opportunity to evaluate locally developed solutions to improve the evidence base of potential strategies for addressing challenges in health care delivery. However, questions remain over the long-term sustainability of this model. Alternative models include low-premium medical insurance schemes and universal health care for managing the cost of NCDs treatment in integrated care [23]. The evidence demonstrating sustainable models for financing integrated chronic care in Africa is still lacking.

### Lesson 4: If implemented well, integrated chronic care can destigmatize HIV and other chronic conditions

Mixing patients with different health conditions to access care from a single point of care was the hall mark of the integration approach in this study. This approach may not appeal to all patients due to stigma related to HIV or other NCDs such as diabetes, as seen when routine services first introduced integrated care. Our findings suggest that adopting a patient centred approach (that is, ensuring that health services respect and are responsive as much as possible to patients’ preferences and needs regarding clinical decisions and their care) may make integrated care more acceptable to patients. Good communication with patients is essential so that patients are aware of changes being made as was done in the research study and understand the reasons for changing from traditional vertical care approaches. Key considerations should also include maintaining patient confidentiality and ensuring adequate care for all patients when implementing integrated care [19].

Additionally, community mobilisation prior to scale-up may increase uptake. In the feasibility study, health facility managers organized community meetings and invited policy makers, clinicians, patient representatives and local leadership to introduce the integrated care model. This may have been perceived as endorsement of the model by the local leadership and may have facilitated patients’ uptake of integrated care. Governments considering scaling up of integrated care can also utilize community mobilization to drive a population health agenda and facilitate community-based integrated screening programmes and case detection for both HIV and NCDs as was done previously in Uganda [24].

### Lesson 5: Decentralizing health care and empowering primary health care facilities will be crucial for integrated care scale up

Lastly, a supportive and flexible regulatory framework allowed health facility managers to scale-up integrated care while awaiting further evidence for policy consideration in Uganda. The regulatory environment empowered health facility managers to implement the model whilst receiving supportive supervision from the Ministry of Health. Regular communication and on-going site visits by policy makers ensured constant guidance to facility managers to always uphold quality health services and patient safety. Notwithstanding, more robust evidence will be essential to guide uptake of any new health policy and changes in clinical practice on integrated care beyond the study sites. Moreover, it will be critical for countries that proceed to scale-up, to monitor and evaluate their integrated care programmes. This knowledge will be essential to expand and sustain the scale-up in sub-Saharan Africa.

## (5) Conclusion

The uptake of the integrated chronic care approach by routine clinical services in Uganda suggests that scale-up of the model is feasible in sub-Saharan Africa. However, successful uptake will require more robust evidence on clinical and economic benefits that accrue from the model for health service providers, patients, and policy makers. This evidence remains limited at present. Finally, strong leadership and addressing systemic challenges inherent in health systems will be critical for successful scale-up of integrated chronic care.
